# Factors Associated With Quality of Life and Pruritus Among Adults With Tinea Infection Attending a Specialty Hospital in Andhra Pradesh, South India: A Cross-Sectional Study

**DOI:** 10.7759/cureus.116903

**Published:** 2026-09-25

**Authors:** Navneetha Vani Ajith Girija, Maria Varghese, Bobby Joseph

**Affiliations:** 1 Internal Medicine, Mersey and West Lancashire Teaching Hospitals NHS Trust, Prescot, GBR; 2 Geriatrics, St. John's Medical College Hospital, Bengaluru, IND; 3 Community Health, St. John's Medical College Hospital, Bengaluru, IND

**Keywords:** cross-sectional studies, india, pruritus, quality of life, tinea

## Abstract

Introduction

Tinea infections, also known as dermatophytosis, are superficial infections that affect the skin, hair, and nails. These infections cause severe pruritus and visible lesions that lead to cosmetic embarrassment, significantly impacting quality of life (QoL). The objectives of the study were to describe the demographic, clinical, and hygiene-related characteristics of adults with tinea infections attending a specialty hospital in Andhra Pradesh, South India and to assess factors associated with QoL and pruritus severity among these patients.

Methods

A cross-sectional study was conducted over three months in the outpatient department of a specialty hospital in Andhra Pradesh, and 269 participants aged 18 years and above, clinically diagnosed with tinea infection, and suffering for at least one month were included. Data was collected using the Dermatology Life Quality Index (DLQI) and the 5-D Itch scale. Statistical analysis was performed using descriptive statistics, chi-square test, independent t-test, correlation and path analysis.

Results

Most participants were aged below 40 years (62.1%) and female (52.8%). The mean DLQI score was 10.97 (SD=4.63), with 42.4% experiencing a large effect on QoL. Significant factors associated with a large DLQI effect (combining very large and extremely large DLQI effect categories) included infection in two or more areas (aOR=2.01, 95% CI: 1.07-3.78; p=0.031), infection duration over six months (aOR=1.90, 95% CI: 1.11-3.23; p=0.019), and recurrent infections (aOR=1.70, 95% CI: 1.00-2.87; p=0.049). The mean 5-D Itch score was significantly associated with multiple affected areas (p<0.001), longer infection duration (p<0.001), infrequent bathing, and sharing personal items like combs and towels (p<0.05). The DLQI and 5-D Itch scores had a significantly strong correlation (r=0.635, p<0.001).

Conclusion

Tinea infections significantly impact patients' QoL, with multiple areas affected, longer infection duration, and recurrent infections being key factors. This study highlights the need for comprehensive interventions to address both medical and psychosocial aspects, improving overall patient well-being.

## Introduction

Tinea infections or dermatophytosis are superficial infections of keratinized tissues such as hair, skin, and nails caused by a specific group of fungi that are antigenically, physiologically, and morphologically related. These fungi are known as dermatophytes or ringworm fungi [1,2].

Dermatophytes can be categorized into three genera: Trichophyton (causing infections on skin, hair, and nails), Epidermophyton (causing infections on skin and nails), and Microsporum (causing infections on skin and hair). Clinically, based on the affected site, these infections are classified as follows: tinea capitis (head), tinea faciei (face), tinea barbae (beard), tinea corporis (body), tinea manus (hand), tinea cruris (groin), tinea pedis (foot), and tinea unguium (nail) [3].

Although *Trichophyton rubrum* has been identified as the most prevalent causal dermatophyte in India, studies from different geographic areas have also found predominance of *Trichophyton mentagrophytes* and *Microsporum audouinii* [3,4].

According to recent studies, the incidence of dermatophytosis has increased across the country in the last decade, particularly in the last 5-6 years [5,6]. The prevalence of the disease in India currently ranges from 36.6 to 78.4% in the general population [7]. With the exception of tinea capitis, dermatophytosis is more common in post-pubertal hosts. Outdoor activities often expose men to hot, humid, and sweaty conditions, which favor the growth of dermatophytes [8]. Men are more commonly affected than women and have a much higher frequency of tinea cruris, tinea pedis, and tinea unguium.

Quality of life (QoL) refers to a person's overall well-being, which encompasses physical health, sexual health, social, psychological, educational, vocational, and financial well-being. The mere presence of any disease can have impact on all the aspects of the QoL [9].

The most common complaint in people with superficial dermatophytosis is pruritus. Although it appears to be a minor symptom, if present in severe form and for an extended period of time, it has a significant impact on the patient's QoL. Another major effect of any skin disease is the cosmetic embarrassment due to visibility of the lesions. This also greatly restricts the patient's social interactions and impairs the QoL [9].

A review of the literature revealed that studies on the impact of tinea infection and associated pruritus on QoL were scarce in India, especially in the southern regions. The objectives of the study were to describe the demographic, clinical, and hygiene-related characteristics of adults with tinea infection attending a specialty hospital in Andhra Pradesh, South India, and to assess factors associated with QoL and pruritus severity among these patients. We believe our study sheds light on the effect of tinea infection on QoL. This in turn will help health professionals and public health physicians recognize the need to address the impact of the disease at a medical and psychosocial level.

## Materials and methods

Study design and study setting

An analytical cross-sectional study was conducted at the outpatient department of a dermatology specialty hospital in Andhra Pradesh, over a period of three months (April 2023 - June 2023).

Study population

The study included consenting participants aged 18 years and above who attended the outpatient department of the specialty hospital and were clinically diagnosed with tinea infection. Participants needed to have been suffering from the disease for at least one month, with or without prior treatment with antifungals. The exclusion criteria were immunocompromised patients and those with comorbidities such as thyroid disorders, pregnancy, psychiatric abnormalities, or any other pre-existing dermatoses.

Purposive sampling was used to include participants meeting the inclusion criteria, and each participant was enrolled only once during the study period; repeat follow-up visits were not included as separate participants.

Study tools

The study tool consisted of questions covering socio-demographic and clinical details of the participants and two standardized questionnaires: the Dermatology Life Quality Index (DLQI) and the 5-D Itch scale.

Socio-Demographic and Clinical Details

Socio-demographic questions covering participants' age, gender, education, occupation, family structure, and clinical details such as comorbidities, clinical diagnosis, areas of body affected, and duration of infection, history of recurrent infection, contact history, completion of treatment during previous infection, and personal hygiene habits like frequency of bathing, sharing combs and towels.

Dermatology Life Quality Index (DLQI)

The first dermatology-specific quality of life questionnaire, published in 1994. DLQI has been used in over 80 countries for a variety of skin diseases and is accessible in over 110 languages [10,11]. The DLQI is made up of ten questions on patients' perceptions of the impact of skin disorders on different aspects of their health-related quality of life. The scoring ranged from 0 to 30 points, and QoL categories were 0-1 (no effect), 2-5 (small effect), 6-10 (moderate effect), 11-20 (very large effect), and 21-30 (extremely large effect). High scores indicated poor QoL. For analytical purposes, and due to small numbers within individual DLQI categories, DLQI scores were grouped into small effect (0-10) and large effect (11-30), with the latter combining very large and extremely large effect categories.

5-D Itch scale

This scale was created as a brief yet multidimensional questionnaire that may be used as an outcome measure. The five dimensions are degree, duration, direction, disability, and distribution of pruritus. In patients with skin illness, liver disease, kidney disease, HIV/AIDS, and burns, the 5-D has shown simplicity of use, content validity, test-retest reliability, internal consistency, and the capacity to identify change in itch over time [12].

All questionnaires were interviewer-administered by the first author. The original English versions of the DLQI and 5-D Itch Scale were used. The first author, who was fluent in the local language, collected all study data using a standardized interview approach. For participants who were not comfortable with English, questions were explained in the local language to ensure comprehension.

Sample size

The sample size was calculated using the formula $n = \frac{Z^2 p q}{d^2}$, assuming a prevalence of 50% for a very large effect on quality of life, a precision of 5%, and a 95% confidence level. In the absence of prior estimates, a prevalence of 50% was assumed to obtain the maximum sample size, resulting in an estimated sample size of 385 participants. Based on outpatient attendance records, the estimated source population during the three-month study period was approximately 800 eligible patients. Each participant was enrolled only once, and repeat follow-up visits were excluded using the hospital's computerized registration system and patient records.

Therefore, finite population correction *N=n/1+n/P*

Where N=finite corrected sample size, n=estimated sample size = 385, P=Fixed population = 800, was applied, resulting in a final sample size of 260 participants.

Analysis

The data were collected using EpiCollect5, entered into Microsoft Excel, and analyzed using IBM SPSS Statistics for Windows, Version 20 (Released 2011; IBM Corp., Armonk, New York, United States). Standard descriptive statistics such as frequency, percentages, and mean with standard deviation were used to describe categorical variables. Bivariate analysis was conducted using the chi-square test of significance, independent t-test, and correlation was done to identify variables with significant association with the outcome of interest. Regression models were run to identify factors independently associated with a large impact on quality of life and 5-D Itch score. An exploratory path analysis was performed to explore the relationships between disease characteristics, pruritus severity, and quality of life.

Ethical statement

Ethical clearance from the Institutional Ethics Committee (Study No 46/2023) and permission from the director of the specialty hospital were obtained prior to commencement of the study. Written informed consent was obtained from all study participants.

## Results

The sociodemographic and clinical characteristics of participants are summarized in Table 1. A total of 269 patients with tinea infection were included, with a mean age of 36.3 ± 13.2 years; 62.1% were aged below 40 years and female patients comprised 52.8% of the study population. Many participants had less than 10 years of education (63.9%), belonged to nuclear families (63.6%) and were gainfully employed (59.5%). Tinea corporis (94.8%) and tinea cruris (78.8%) were the most common clinical types. Multi-site infection was seen in 76.2%, and infection duration more than six months was seen in 34.9% of participants. Contact history was reported by 54.6%, and recurrent infection in 39.8%. Regarding personal habits, 76.2% reported no tobacco or alcohol use. A majority (56.9%) did not bathe daily, while sharing of combs and towels was seen in 64.3% and 45.4% of participants, respectively.

**Table 1:** Socio-demographic and clinical characteristics of participants (N=269) Data is presented in the form of frequency (N) and percentage (%).

Variable	Categories	N (%)
Age	≤ 40 years	167 (62.1)
	40-59 years	85 (31.6)
	≥ 60 years	17 (6.3)
Sex	Male	127 (47.2)
	Female	142 (52.8)
Years of education	≤ 10 years	172 (63.9)
	> 10 years	97 (36.1)
Occupation	Gainfully employed (business, factory workers, farmers, labourers, working with livestock, others)	160 (59.5)
	Not gainfully employed	109 (40.5)
Religion	Hindu	184 (68.4)
	Others	85 (31.6)
Family type	Nuclear	171(63.6)
	Others	98 (36.4)
Comorbidities	Diabetes mellitus	20 (7.4)
	Hypertension	18 (6.7)
	Others	6 (2.3)
	Absent	225 (83.6)
Clinical diagnosis (not mutually exclusive)	Tinea corporis	255 (94.8)
	Tinea cruris	212 (78.8)
Areas of body affected with infection	Single area	64 (23.8)
	≥ Two areas	205 (76.2)
Duration of infection	> 6 months	94 (34.9)
	≤ 6 months	175 (65.1)
Contact history	Yes	147 (54.6)
	No	122 (45.4)
Habits	Tobacco and alcohol	8 (3.0)
	Alcohol only	10 (3.7)
	Tobacco use only	36 (13.4)
	Others	10 (3.7)
	None	205 (76.2)
Recurrent infection	Yes	107 (39.8)
	No	162 (60.2)
Completion of entire course of treatment during previous infection?	Yes	16 (5.9)
	No	91 (33.9)
	Not applicable	162 (60.2)
Frequency of bathing	Daily	116 (43.1)
	Less than daily	153 (56.9)
Do you share combs	Yes	173 (64.3)
	No	96 (35.7)
Do you share towels	Yes	122 (45.4)
	No	147 (54.6)

The mean (SD) DLQI score was 10.97 (4.63), indicating an above moderate effect on QoL with about 42% of participants having a very large to extremely large effect (Table 2). The mean 5D pruritus score was 16.63 ± 4.35, reflecting moderate pruritus severity.

**Table 2:** DLQI scores of the participants with interpretation (N=269) Data is presented in the form of frequency (N) and percentage (%). DLQI: Dermatology Life Quality Index

Variable	Categories	N (%)
DLQI interpretation	No/small effect (0-5)	81 (30.1)
	Moderate effect (6-10)	74 (27.5)
	Very large to extremely large effect (11-30)	114 (42.4)

Table 3 shows the association of DLQI and various sociodemographic and clinical factors. Participants with involvement of two or more body areas, individuals with infection lasting more than six months and those with recurrent infections had a significantly higher odds of having a large DLQI effect. 

**Table 3:** Association of DLQI with sociodemographic and clinical variables (N=269) *df: 1; OR: Odds Ratio; CI: Confidence Interval; DLQI: Dermatology Life Quality Index

Variables	DLQI categories		p-value	Chi-square value (χ²)*	OR (95% CI)
	Large effect N=114	Small effect N=155			
Age					
≤ 40 years	69 (41.3)	98 (58.7)	0.65	0.2033	0.89 (0.54-1.47)
> 40 years	45 (44.1)	57 (55.9)			-
Gender					
Female	63 (44.4)	79 (55.6)	0.49	0.4863	1.18 (0.73-1.93)
Male	51 (40.2)	76 (59.8)			-
Education					
≤ 10 years	77 (44.7)	95 (55.2)	0.29	1.1141	1.31 (0.79-2.18)
> 10 years	37 (38.1)	60 (61.9)			-
Occupation					
Gainfully employed	65 (40.6)	95 (59.4)	0.48	0.4976	0.84 (0.51-1.37)
Not gainfully employed	49 (45.0)	60 (55.0)			-
Religion					
Hindu	73 (39.7)	111 (60.3)	0.19	1.7452	0.71 (0.42-1.18)
Others	41 (48.2)	44 (51.8)			-
Family type					
Nuclear	68 (39.8)	103 (60.2)	0.25	1.3125	0.74 (0.45-1.23)
Others	46 (46.9)	52 (53.1)			-
Comorbidities					
Present	17 (38.6)	27 (61.4)	0.58	0.3018	0.83 (0.43-1.61)
Absent	97 (43.1)	128 (56.9)			-
Habits					
Yes	27 (42.2)	37 (57.8)	0.97	0.0013	0.99 (0.56-1.75)
No	87 (42.4)	118 (57.6)			-
Areas affected					
Two areas or more	96 (46.8)	109 (53.2)	0.008	6.9876	2.25 (1.22-4.14)
Single area	18 (28.1)	46 (71.9)			-
Duration of infection					
> 6 months	52 (55.3)	42 (44.6)	0.002	9.9078	2.25 (1.35-3.76)
≤ 6 months	62 (35.4)	113 (64.6)			-
Recurrent infection					
Yes	54 (50.5)	53 (49.5)	0.029	4.7597	1.73 (1.06- 2.84)
No	60 (37.0)	102 (63.0)			-

A multivariable binary logistic regression analysis (Table 4) was performed to identify factors independently associated with a large impact on QoL. After adjustment for potential confounders, involvement of two or more body areas (aOR = 2.01, 95% CI: 1.07-3.78; p = 0.031) infection duration greater than six months (aOR = 1.90, 95% CI: 1.11-3.23; p = 0.019), and recurrent infection (aOR = 1.70, 95% CI: 1.00-2.87; p = 0.049) remained independently associated with a large impact on QoL.

**Table 4:** Multivariable logistic regression of factors associated with impact of DLQI among adults with tinea infection (N = 269) CI: Confidence Interval; Ref: Reference; DLQI: Dermatology Life Quality Index

Variable	Category	DLQI	95% CI	p-value
		Adjusted OR (aOR)		
Age	≤40 years	1.01	0.60–1.70	0.976
	>40 years	Ref	-	-
Sex	Female	1.38	0.82–2.32	0.227
	Male	Ref	-	-
Areas affected	Two or more body areas	2.01	1.07–3.78	0.031
	Single body area	Ref	-	-
Duration of infection	>6 months	1.90	1.11–3.23	0.019
	≤6 months	Ref	-	-
Recurrent infection	Yes	1.70	1.00–2.87	0.049
	No	Ref	-	-
Model statistics: Overall model χ² = 19.1, df = 5, p = 0.002; Nagelkerke R² = 0.069.				

Table 5 presents the associations of 5D Itch scores with sociodemographic and clinical variables. Higher pruritus scores were significantly associated with age more than 40 years (17.69 ± 4.47, p = 0.002), involvement of two or more body areas (17.41 ± 4.14, p < 0.05), and infection duration exceeding six months (18.24 ± 3.96, p < 0.05). Significant differences were also observed with bathing frequency, sharing of combs, and sharing of towels (all p < 0.05). Participants experiencing a larger impact on quality of life had higher pruritus scores (19.93 ± 2.89) compared with those with a smaller impact (14.21 ± 3.58, p < 0.001). However, multiple linear regression (Table 6) showed that age more than 40 years, infection duration more than six months, and involvement of two or more body areas were independently associated with higher 5D Itch scores.

**Table 5:** Association of the 5D Itch score with socio-demographic and clinical variables (N=269) *Independent t-test SD: Standard deviation

Variables	Categories	5 D score Mean (SD)	T-statistic	p-value*
Age	≤ 40 years	15.99 (4.15)	3.1653	0.002
	> 40 years	17.69 (4.47)		
Areas affected	≥ Two areas	17.41 (4.14)	5.6122	<0.001
	Single area	14.10 (4.05)		
Duration of infection	> 6 months	18.24 (3.96)	4.7472	<0.001
	≤ 6 months	15.76 (4.31)		
Frequency of bathing	Daily bathing	15.70 (4.29)	3.0903	0.002
	Less than daily	17.33 (4.28)		
Share combs	Yes	17.06 (4.40)	2.2010	0.027
	No	15.85 (4.17)		
Share towels	Yes	17.43 (4.50)	2.7747	0.006
	No	15.97 (4.12)		

**Table 6:** Multiple linear regression of factors independently associated with 5-D Itch score among adults with tinea infection (N=269)

Variable	Category	B (95% CI)	p-value
Age	>40 years vs. ≤40 years	1.30 (0.32-2.29)	<0.010
Duration of infection	>6 months vs. ≤6 months	1.63 (0.60-2.66)	0.002
Areas affected	Two or more areas vs. Single area	2.89 (1.76-4.02)	<0.001
Recurrent infection	Yes vs. No	0.10 (−0.88-1.07)	0.845
Bathing frequency	Less than daily vs. Daily	0.94 (−0.08-1.96)	0.071
Sharing combs	Yes vs. No	0.74 (−0.27-1.75)	0.152
Sharing towels	Yes vs. No	0.69 (−0.35 to 1.72)	0.193

Pearson’s correlation showed a strong positive correlation between DLQI and 5D Itch scores (r=0.635, p < 0.001), indicating that higher pruritus severity was associated with a larger effect on the quality of life.

Path analysis (Table 7 and Figure 1) was conducted to examine the direct and indirect effects of infection-related characteristics such as the number of areas affected, duration of infection, and recurrent infection on quality of life (DLQI), with itch severity (5D score) as the mediator. The number of areas affected showed a significant indirect effect on DLQI through itch severity (p < 0.05), while its direct effect on DLQI was not significant, indicating that its impact on quality of life was primarily mediated by itch severity. Similarly, the duration of infection demonstrated a significant negative indirect effect on DLQI via itch severity (p < 0.05), with no significant direct effect on DLQI. In contrast, recurrent infection showed a significant direct effect on DLQI (p < 0.05), whereas its indirect effect through itch severity was not significant, indicating a predominantly direct impact on QoL.

**Table 7:** Path analysis showing direct, indirect, and total effects *p<0.05 Duration of infection was coded as 1 = >6 months and 2 = ≤6 months. Therefore, negative coefficients indicate higher effects among participants with infection duration >6 months.

Variables	Direct Effect	Indirect Effect	Total Effect
No. of areas affected → QoL	-0.117	3.325*	3.209*
Duration of infection → QoL	-0.004	-2.480*	-2.485*
Recurrent infection → QoL	-1.067*	-0.500	-1.567*

**Figure 1:**
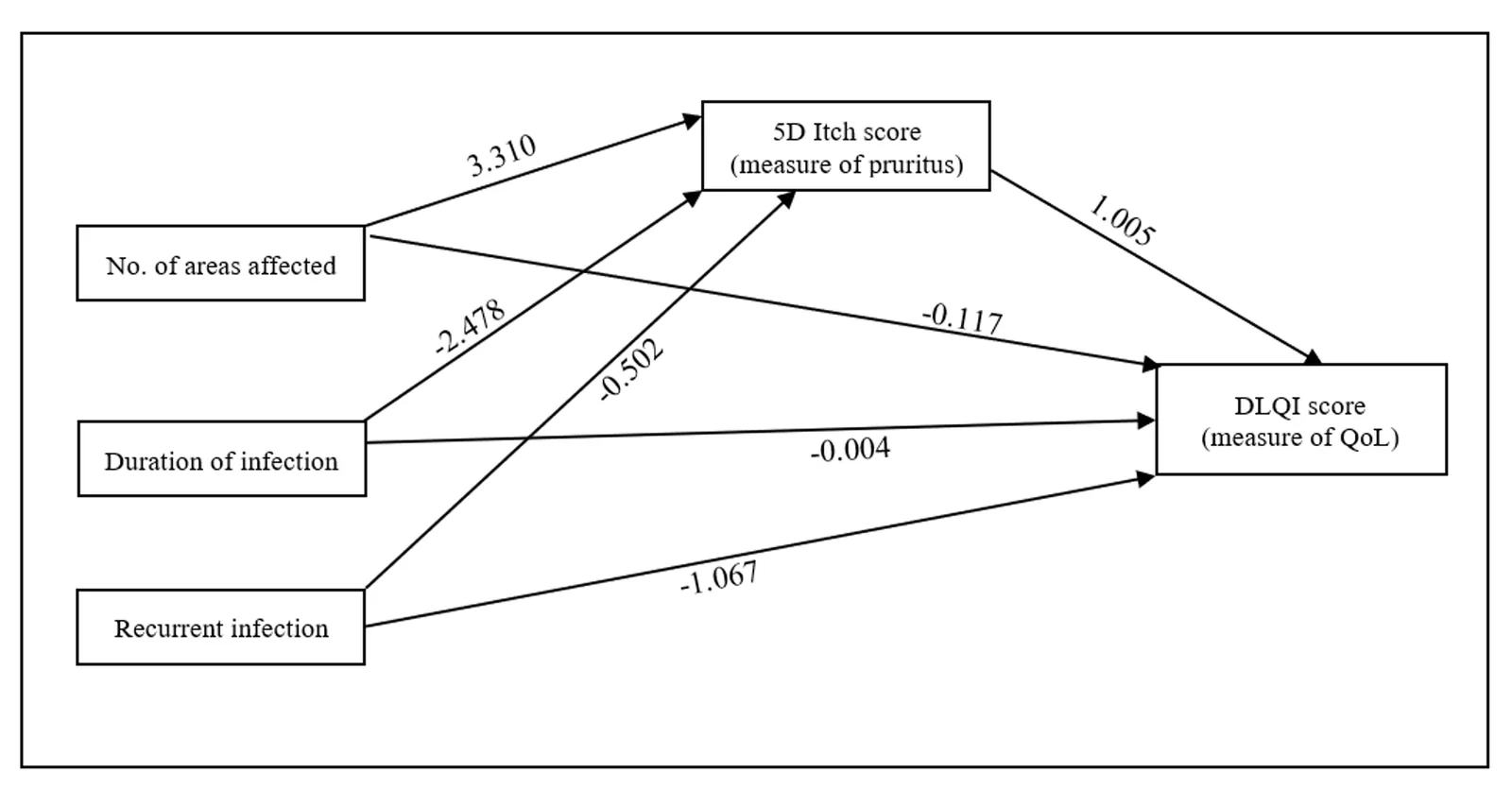
Path analysis indicating the causal pathway between the number of areas affected by tinea, duration of infection and recurrent infection with quality of life through pruritus

## Discussion

The socio-demographic profile of this study was predominantly young to middle-aged adults (62.1%), with a slight female predominance (52.8%) and lower educational status (63.9%), reflecting trends observed in other studies [9,11,13]. Tinea corporis and tinea cruris were the most common clinical types of dermatophytosis seen in this study, and a substantial proportion of participants (76.2%) had infections involving multiple areas of the body, a pattern also reported in other studies [9,14-16]. Hot and humid environments are ideal for dermatophytes to thrive, making many parts of India particularly susceptible to tinea corporis and cruris. Areas of the body like the trunk and groin are more likely to remain moist due to sweating and tight clothing, facilitating fungal growth and spread [6,17]. A high proportion of contact history (54.6%) and sharing of personal items such as combs (64.3%) and towels (45.4%) seen in this study further aligns with established behavioral risk factors and lack of awareness about transmission and hygiene practices [18-20].

In this study, the mean (SD) DLQI score of 10.97 (4.63) indicated an above-moderate impact on QoL, and about nearly half of the participants (42.4%) reported “very large” to “extremely large effect” on QoL. These findings were similar to studies by other authors, including Doshi et al. and Rathwa et al., among others [16,19,21,22]. This study also observed that a large effect on DLQI was strongly associated with chronicity, recurrent disease, and an increased number of affected areas, findings that are consistent with evidence that longer disease duration and more body surface involvement increase psychosocial distress [11,13,16,17] and lead to cosmetic embarrassment [9,16].

The mean (SD) 5D Itch score in this study was 16.63 (4.35). Age more than 40 years, involvement of two or more body areas, and infection duration exceeding six months and was independently associated with higher itch scores. Similar findings were noted in other studies as well [9,11,13]. Due to age-related changes in skin barrier function and immune response, a higher prevalence and intensity of itching is often seen in older adults [23]. An extensive infection, lasting a long time, exposes a larger area of skin to inflammation and fungal antigens, resulting in increased nerve stimulation and itching [9]. 

A strong positive correlation (r=0.635, p < 0.001) between pruritus severity and DLQI was noted in this study. This is in line with other studies, emphasizing pruritus as one of the most distressing symptoms [9,11,24]. While multi-site involvement of infection has been independently linked to poor QoL [9], poor QoL is also a consequence among participants with higher itching severity, leading to functional and emotional impairment. The exploratory path analysis illustrates that the association between multi-site involvement and poorer QoL was mediated through pruritus, with a significant indirect effect but no significant direct effect.

This study has certain limitations. Its cross-sectional design and being a single-center study with purposive sampling could have prevented causal interpretations and limited the generalizability of the findings. Clinical diagnosis without mycological confirmation could have introduced misclassification, and reliance on self-reporting of symptoms and behaviors could have led to recall bias. In addition, previous treatment history, including topical corticosteroid use, was not assessed in detail. The path analysis was exploratory and does not establish causality.

## Conclusions

The findings of this study highlight that chronicity of disease, multi-site involvement, and pruritus were associated with poorer QoL among adults with tinea infection. Beyond physical distress, the condition also contributes to psychosocial burden, affecting daily functioning, emotional well-being, and social interactions.

The study also underscores the need for comprehensive management of the disease that extends beyond antifungal therapy alone. Emphasis should be placed on effective symptom control, consistent treatment adherence, and targeted health education to reduce transmission and improve hygiene. Addressing both medical and psychosocial aspects may help reduce disease persistence, prevent recurrence, and ultimately improve overall QoL.
